# Immediate and Long-Term Radiopacity and Surface Morphology of Hydraulic Calcium Silicate-Based Materials

**DOI:** 10.3390/ma15196635

**Published:** 2022-09-24

**Authors:** Goda Bilvinaite, Saulius Drukteinis, Vilma Brukiene, Sivaprakash Rajasekharan

**Affiliations:** 1 Institute of Dentistry, Faculty of Medicine, Vilnius University, Zalgirio 115, LT-08217 Vilnius, Lithuania; 2 Department of Paediatric Dentistry, School of Oral Health Sciences, Ghent University, B-9000 Ghent, Belgium

**Keywords:** calcium silicate, endodontics, hydration, microscopy, radiopacity, surface structure

## Abstract

The present study aimed to evaluate and compare the radiopacity and surface morphology of AH Plus Bioceramic Sealer (AHPB), Bio-C Sealer (BIOC), Biodentine (BD), BioRoot RCS (BR), Grey-MTAFlow (GMF), White-MTAFlow (WMF), TotalFill BC Sealer (TF), and TotalFill BC Sealer HiFlow (TFHF) at different time moments—30 min, 24 h, and 28 days. Ten specimens of each material were prepared according to the ISO-6876:2012 standard and radiographed next to an aluminum step wedge using a digital sensor. The specimens were stored in a gelatinized Hank’s balanced salt solution at 37 °C between assessments. The mean grayscale values of each specimen were converted into equivalent aluminum thickness by a linear regression model. Characterization of the surface morphology was performed by using a scanning electron microscope at ×4.0k and ×10.0k magnifications. The radiographic analysis revealed that all the tested materials exceeded the ISO-specified limit of 3 mm Al, with the highest radiopacity presented by AHPB and the lowest by BD. None of the tested materials demonstrated considerable variances between the 30 min and the 24 h radiopacity level (*p <* 0.05), and statistically significant long-term radiopacity changes were exhibited by BR, TFHF, and TF (*p >* 0.05). All the specimens demonstrated a common feature of limited precipitate formation, with numerous unreacted particles still presented on the surface after 24 h, whereas the particle rearrangement and the deposition of precipitates were clearly observed after 28 days.

## 1. Introduction

Hydraulic calcium silicate cements (HCSCs), also known as bioceramics, constitute a group of materials that can be considered as a breakthrough in endodontics. These materials set hard in the presence of moisture and thus can be successfully used for a wide range of procedures, including vital pulp therapy, regenerative endodontics, root canal obturation, perforation repair, and endodontic surgery [1]. The advantageous physical, chemical, and biological properties, which have been extensively investigated for more than three decades, ultimately led to HCSCs being the material of choice in modern clinical endodontics [2]. However, some concerns have been raised about the HCSCs’ radiopacity, as previous studies demonstrated that HCSCs are less radiopaque than epoxy resin-based AH Plus sealer (Dentsply Sirona, Ballaigues, Switzerland), which is considered to be the gold standard [3,4].

The radiopacity of endodontic materials has been widely acknowledged to be of particular significance for a clear distinction between the filling material and the surrounding anatomic structures on periapical radiographs or cone-beam computed tomography (CBCT) [5,6]. Even though radiographic images and CBCT do not provide high sensitivity in small pore detection, and thus the assessment of filling homogeneity [7], these methods are the only ones clinically available to evaluate the length and overall homogeneity of endodontic fillings after root canal obturation. Therefore, the International Organization for Standardization (ISO) has established 3 mm aluminum (Al) thickness as the minimal value of radiopacity for all root canal filling materials at the thickness of 1 mm [8]. In order to meet this requirement, various radiopacifying agents must be incorporated in HCSCs, as HCSCs with no additives would otherwise have an intrinsic radiopacity of only 0.86–2.02 mm Al [9].

Bismuth oxide was a commonly used radiopacifier in the first generation of HCSCs, including mineral trioxide aggregate (MTA) [10]. Even though this radiopacifying agent can be still found in some new MTA formulations, such as Grey-MTAFlow (Ultradent Products Inc., South Jordan, UT, USA), alternative radiopacifiers, mainly zirconium or tantalum oxides, have lately been added to HCSCs, aiming to prevent the tooth discoloration associated with bismuth oxide and its reactivity [11]. Previous research confirmed that zirconium and tantalum oxides maintain color stability and are leached in minimal quantities, while only 8% of bismuth oxide remains in the initial form after 28 days of hydration [12]. However, the main drawback of alternative radiopacifiers has been related to a lower atomic number and molecular weight [13], which provides weaker photoelectric absorption and X-ray scattering as compared to bismuth oxide. Therefore, attempts to overcome this limitation and achieve adequate radiopacity have led to different amounts of radiopacifying agents being added to HCSCs [2].

The diversity of chemical composition and proportions, which typically are not detailed by manufacturers, has been mainly associated with a wide-ranging scale of radiopacity values observed in the scientific literature [14]. However, the choice of imaging system and exposure parameters was also reported to exert an influence on the radiopacity level [15], leading to intra-material variations up to 25% [16] and thus raising difficulties in making a conclusive evaluation and comparison of the published data. Moreover, the radiopacity of HCSCs is routinely assessed after the complete setting of the material in time periods of 24 h to 28 days, whereas the clinically radiographic images are obtained immediately after the endodontic treatment is finished. Considering the primary porosity of HCSCs, which depends on the powder-packing characteristics and has a tendency to decrease due to progressive hydration and precipitate formation [17,18], the initial radiopacity can be expected to be lower than declared by the manufacturers or previous studies. This approach is closely related to the chemical kinetics, which has been observed to achieve the highest rates within the first 24 h of material application and then to continue at significantly lower activity levels [19], as the formation of calcium silicate hydrate (CSH) and precipitation potentially reduce the available surface of the reacting particles [20]. However, the available data on how chemical reactions may influence the immediate and long-term radiopacity of HCSCs are still limited.

Clinicians should be aware of radiopacity and the changes of the endodontic materials that they use, as it may influence the interpretation of filling homogeneity and overall quality, particularly in areas with smaller amounts of the material. Therefore, the present study aimed to evaluate and compare the radiopacity and surface morphology of the most common HCSCs at different time moments—30 min, 24 h, and 28 days. The tested hypothesis was that the time elapsed after the application of HCSCs has a significant impact on the radiopacity and surface structure.

## 2. Materials and Methods

The materials tested in this study included AH Plus Bioceramic Sealer (AHPB; Dentsply Sirona, Ballaiques, Switzerland), Bio-C Sealer (BIOC; Angelus, Londrina, Brazil), Biodentine (BD; Septodont, Saint-Maur-des-Fosses, France), BioRoot RCS (BR; Septodont, Saint-Maur-des-Fosses, France), Grey-MTAFlow (GMF; Ultradent Products Inc., South Jordan, UT, USA), White-MTAFlow (WMF; Ultradent Products Inc., South Jordan, UT, USA), TotalFill BC Sealer (TF; FKG, La Chaux-de-Fonds, Switzerland), and TotalFill BC Sealer HiFlow (TFHF; FKG, La Chaux-de-Fonds, Switzerland). The composition of the HCSCs used is described in Table 1.

### 2.1. Assessment of Radiopacity

The radiopacity was evaluated according to the ISO-6876:2012 standard. Ten specimens of each material were prepared as described in Table 1. Circular plastic molds 10 mm in diameter and 1 mm in thickness were used to shape and standardize the specimens. The first radiographs were obtained after the elapse of 30 min from material preparation and initial setting. The specimens were placed next to 98.5% aluminum step wedge, graduated from 2 to 14 mm (1 mm increment per step) and radiographed using the Kodak RVG 5100 digital sensor (Kodak Co., Rochester, NY, USA). The exposure parameters were set at 65 kV, 7 mA, 0.2 s, a 30 cm source-to-object distance, and 0° vertical and horizontal angulations. Specimens with pores or cracks seen on the initial radiograph were discarded and replaced, followed by a new radiograph. 

The approved specimens were allowed to set in gelatinized Hank’s balanced salt solution (HBSS) for 24 h at 37 °C and 5% CO2 atmosphere. The HBSS was gelatinized by using 20% porcine gelatin in an attempt to avoid material washout. The second radiographs after the incubation period of 24 h were obtained as described above. The specimens were subsequently stored in freshly prepared gelatinized HBSS for 28 days at 37 °C and then radiographed for the third and last time. 

All the radiographs were saved in a 16-bit DICOM format and analyzed by a single-blinded examiner using ImageJ v.1.51 software (National Institutes of Health, Bethesda, Rockville, MD, USA). The mean grayscale values of a standardized 76 mm^2^ area in the center of each specimen were calculated and then converted into equivalent aluminum thickness (mm Al) using the linear regression model with a coefficient of determination (R2) equal to 0.986.

### 2.2. Characterization of Surface Morphology

The surface morphology was assessed using a scanning electron microscope (SEM), the Hitachi SU-70 (Hitachi, Tokyo, Japan). Two sets of each HCSC, containing 3 cylindrical 10 × 1 mm specimens per set, were prepared as described in Table 1 and stored in gelatinized HBSS for 24 h (1st set) and 28 days (2nd set). After the specified time period, the specimens were dried in a vacuum desiccator without coating, attached to an aluminum stub and examined under SEM at ×4.0k and ×10.0k magnifications.

### 2.3. Statistical Analysis

The minimum sample size was calculated using G*Power v.3.1.9.7 software (Heinrich Heine, Dusseldorf, Germany) followed by an α error probability of 0.05 and a power (1-β error probability) of 0.95. The required size of 2 specimens per group was determined. 

Statistical analysis was performed using RStudio v.4.1.1 software (RStudio Inc., Boston, MA, USA). The assumption of normality was assessed and confirmed with a Shapiro–Wilk test. One-way analysis of variance (ANOVA), followed by Tukey’s test, was selected for comparisons of the intra-group radiopacity values, with a significance level set at 5%. The differences between materials that were similar by name (TF versus TFHF, and GMF versus WMF) were determined using the independent samples *t*-test after verification of the homogeneity of variance by the two-variances F-test.

## 3. Results

### 3.1. Radiopacity

The radiographic analysis revealed that all the tested HCSCs exceeded the ISO-specified limit of 3 mm Al. The lowest radiopacity was presented by BD (3.34–3.75 mm Al) and the highest by AHPB (10.82–11.26 mm Al). The mean radiopacity values of each material expressed in mm Al equivalents are summarized in Table 2.

The positive correlation between the radiopacity and the time elapsed after the application was observed for all the HCSCs used. However, there were no considerable variances between the 30 min and the 24 h radiopacity values (*p* < 0.05), and statistically significant long-term radiopacity changes were exhibited only by BR, TFHF, and TF (total increases of 0.89, 0.98, and 1.10 mm Al in 28 days, respectively). The pairwise comparison of materials similar by name also revealed the radiopacity being significantly different between GMF and WMF in all time periods, while TF and TFHF were shown to be similarly radiopaque.

### 3.2. Surface Morphology

**Figure 1:**
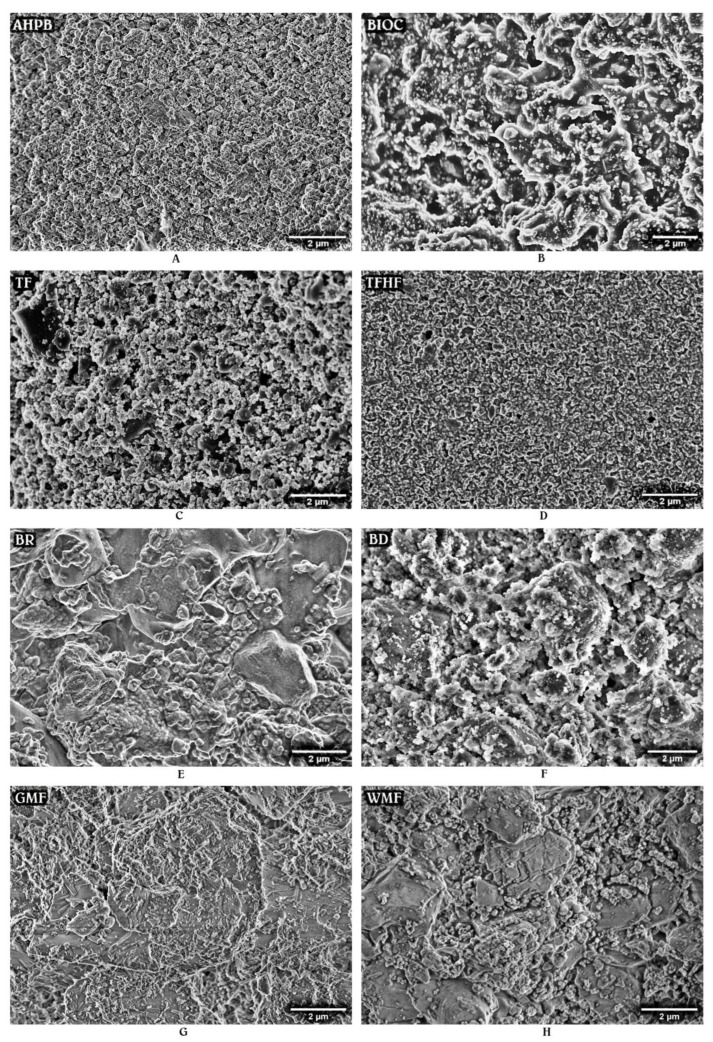
Representative SEM images (×10.0k magnification) of AHPB (**A**), BIOC (**B**), TF (**C**), TFHF (**D**), BR (**E**), BD (**F**), GMF (**G**), and WMF (**H**) after the storage in HBSS for 24 h at 37 °C.

The SEM analysis demonstrated that HCSCs possess a different surface structure. Figure 1 presents the primary micrographs of the materials stored in HBSS for 24 h. The most homogeneous microstructure was observed for AHPB and TFHF, followed by TF. In general, all the premixed HCSCs at this time period exhibited a densely packed surface with respect to a small particle size and regular shape (Figure 1A–D), whereas the mixed materials were composed of large crystallites embedded in a matrix and surrounded by smaller spherical and angular particles (Figure 1E–H). The distribution of these particles and crystallites was observed to be irregular, with the exception of BD, which was mixed in the amalgamator and thus resulted in more homogeneous and uniform particle dispersion (Figure 1F). However, all the tested HCSCs demonstrated a common feature of limited precipitate formation with numerous unreacted particles still presented on the surface after 24 h of aging.

**Figure 2:**
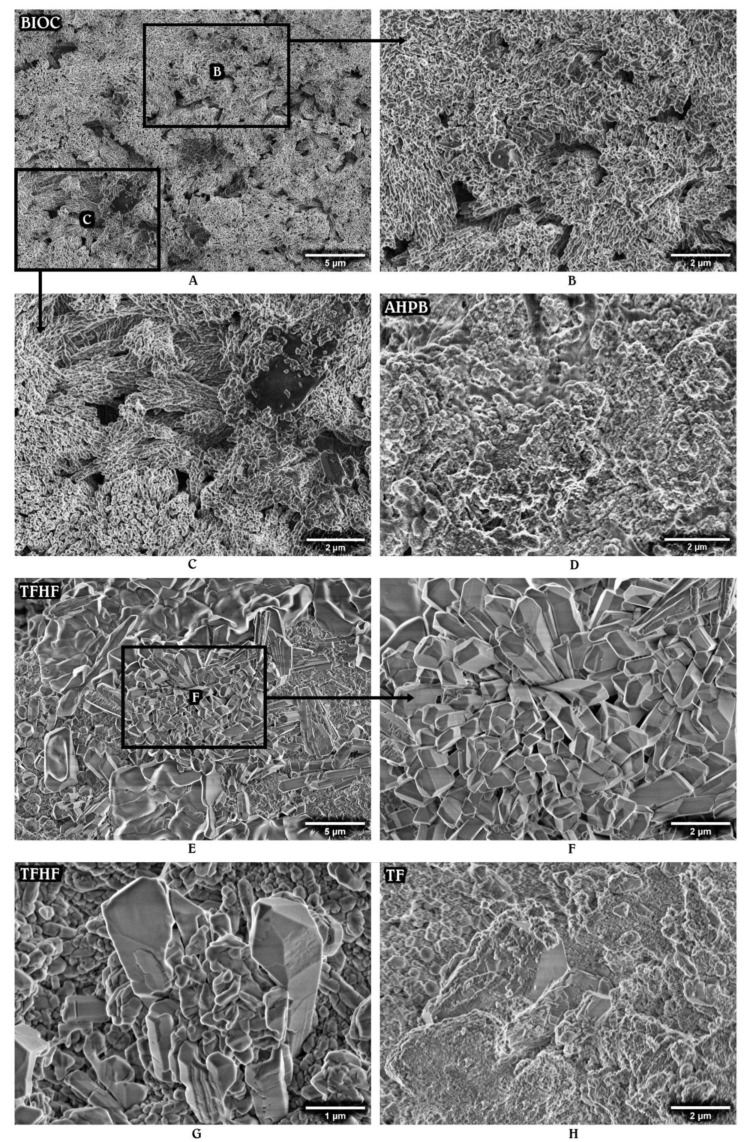
Representative micrographs of premixed hydraulic calcium silicate-based materials: BIOC (**A**–**C**), AHPB (**D**), TFHF (**E**–**G**), and TF (**H**) after 28 days of aging in HBSS. Magnification set at ×4.0k and ×10.0k.

**Figure 3:**
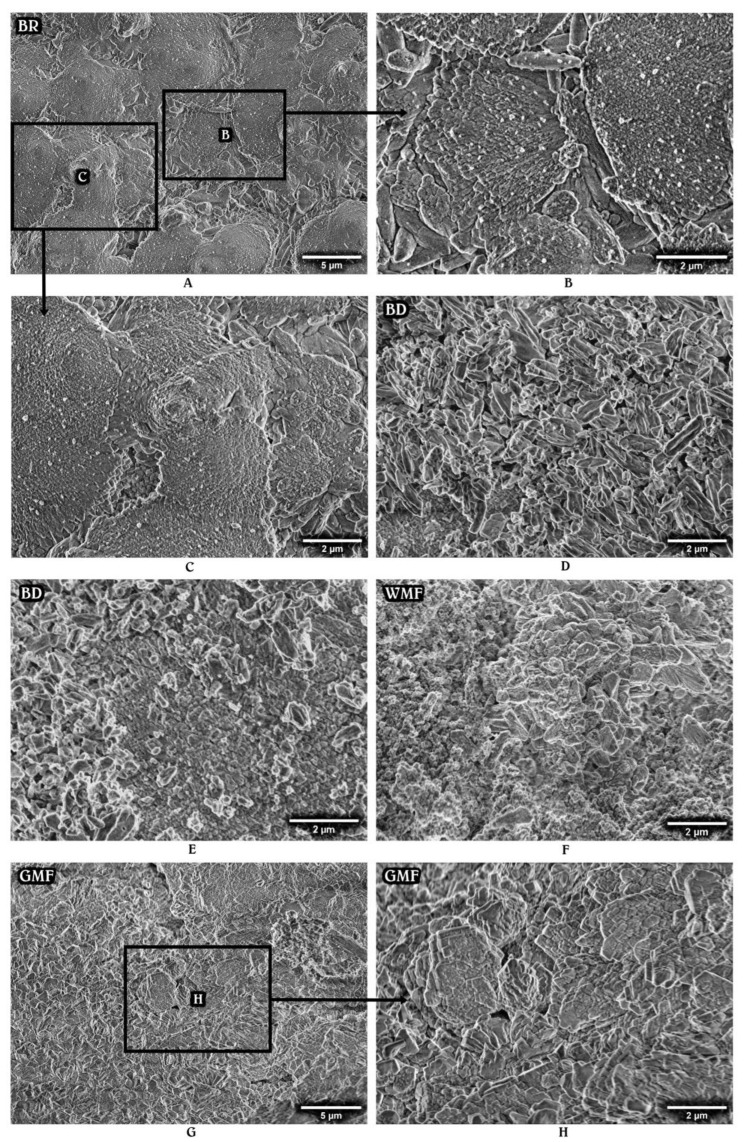
Representative SEM images (×4.0k and ×10.0k magnification) of mixed hydraulic calcium silicate-based materials: BR (**A**–**C**), BD (**D**,**E**), WMF (**F**), and GMF (**G**,**H**) after the storage in HBSS for 28 days.

Secondary micrographs of the set materials stored in HBSS for 28 days are shown in Figure 2 and Figure 3. The surface structure changes, mainly associated with particle rearrangement and the deposition of hydration products, were observed for all the HCSCs. However, the interaction between the tested material and HBSS appeared to have a different impact on the nucleation and growth processes. 

The most notable changes were seen in TFHF, BR, and BIOC. The latter material exhibited a hair-like superficial layer composed of spherical and rod-shaped particles that were arranged in variously oriented bundles (Figure 2A–C). TFHF demonstrated a clear deposition of euhedral prismatic and cubical crystalline structures, formed over a tightly packed mass of fine particles and partly covered by amorphous gel-like substance in some areas (Figure 2E–G). BR displayed the precipitation of mostly acicular-prismatic structures that were oriented parallel to their long axis and clustered in separate regions radially growing along the surface (Figure 3A–C). 

The other tested HCSCs had no specific particle arrangement or appearance of precipitates. BD demonstrated deposits of varying size and shape scattered over a densely packed matrix (Figure 3D,E), whereas AHPB, TF, WMF, and GMF showed multiple aggregates nucleated on the surface. In general, the SEM analysis revealed that all the materials exhibited more solid, compact, and stable surface structure after 28 days of incubation in HBSS, as compared to the initial micrographs.

## 4. Discussion

Regardless of recent advances in the diagnostic tools across the medical field, two-dimensional periapical radiographs combined with a growing use of CBCT remain a standard method for determining the state of periapical tissues and the quality of endodontic treatment [21]. An appropriate radiopacity is thus a highly desirable feature for all endodontic materials to be clearly visible on a radiograph and easily distinguishable from the surrounding anatomical structures [22]. The initial radiopacity, which enables the evaluation of filling quality immediately after the treatment, highly depends on the atomic number of elements that constitute the material [15]. The atomic number is equivalent to the number of protons, which determine the electrical charge of the nucleus and thus the force binding an electron to the orbital [23]. According to the explanation of photoelectric effect, the more tightly an electron is bound to its orbital, the more X-ray energy is absorbed [23]. Therefore, bismuth, having an atomic number of 83, is generally acclaimed to be a more radiopaque element as compared to zirconium and tantalum with atomic numbers of 40 and 73, respectively. This concept supports the statistically significant differences obtained between the initial radiopacity values of GMF and WMF, where bismuth oxide being added to GMF formulation as a radiopacifier has been replaced by tantalum oxide in WMF to avoid potential tooth discoloration [24]. However, the explanation of the varying radiopacity by the atomic number solely is valid only for materials similar by all other characteristics except the radiopacifying agent. Otherwise, the initial X-ray attenuation of the specimens standardized to 1 mm thickness may also be influenced by the chemical composition and its proportions [15], consequently providing a wide-ranging scale of radiopacity values even for HCSCs containing the same radiopacifier. In fact, the percentage of radiopacifying agent, along with other compounds of a high mass attenuation coefficient, can be considered as a decisive factor that has imparted superior radiopacity values to bismuth-free AHPB, BIOC, BR, TF, and TFHF as compared to GMF. 

The expectation of radiopacity changes within the first 24 h of material application mainly arose from the previously investigated setting kinetics of HCSCs. It is well known that HCSCs with the hydrophilic nature set via the hydration of di- and tri-calcium silicates [25]. The process typically begins on the release of hydroxyl (OH¯) and calcium (Ca^2+^) ions, with a consequent break of the covalent siloxane bonds (Si-O-Si) and the Ca^2+^ linkage to the silanol group (Si-OH), which results in a colloidal gel, called calcium silicate hydrate (CSH) [20]. The radially growing matrix of CSH covers the unhydrated cement particles, aggregating them together, and forms a less porous solidified structure that implies a considerably lower diffusion coefficient [20]. For this reason, hydration reactions, which may continue at slow rates for months, are generally assumed to reach their highest activity level within the first 24 h [19]. However, no significant radiopacity variances were observed in the present study after the elapse of 24 h, leading to considerations that the initial structural changes arising from the hydration process have a limited influence on the radiopacity of HCSCs. On the other hand, more time may have been required for the tested materials to complete the main reactions of the hydration and hardening phases as SEM analysis revealed numerous unreacted particles still presented on the surface at the 24 h time point. 

The rate of setting reactions apparently depends on many factors, of which the material composition, as well as the particle morphology and packing characteristics, remains uncontrollable and may be associated with differences in chemical kinetics [26,27]. In general, HCSCs with higher rates of setting reactions are expected to have a faster formation of CSH and thus result in the reduced solubility and washout potential, along with a more tightly packed material matrix transmitting less X-ray photons to the detector plate [15,20]. This concept presumably explains a varying increase in the radiopacity values after the initial 24 h setting period, in which the highest positive changes were determined for TF, TFHF, and GMF (with increases of 0.47, 0.38, and 0.35 mm Al, respectively). Additionally, GMF had a more prominent increase in radiopacity as compared to WMF (0.35 mm Al versus 0.04 mm Al), leading to considerations that bismuth, participating in the hydration process and taking silicon lattice sites in the CSH structure, may possess a lower leaching fraction due to a more efficient incorporation into the matrix than the chemically inert zirconium oxide [28]. 

More evident radiopacity changes of tested HCSCs were determined after 28 days of aging. As the atomic number of elements is known to remain constant despite physical and chemical interactions, the long-term radiopacity increase may be solely associated with precipitates of calcium minerals, such as hydroxyapatite and its precursors. The precipitation ability of HCSCs has been previously confirmed by a number of studies [29], and is currently supported by SEM analysis, which revealed the presence of surface deposits after 28 days of storage. Even though the chemical characterization of the specimens has not been performed in the present study, the clearly visible precipitates, particularly in the BIOC, TFHF, and BR materials, might be suggested as being different types of calcium phosphate, which typically forms on the HCSC surface as a result of calcium and phosphate ion absorption to the Si-OH group and may have a varying structure from stable crystalline, as noticed in TFHF, to nearly amorphous, as displayed by AHPB [25,30]. The process commonly occurs within the first few days of material application [26], explaining the limited precipitation noticed for all the tested materials after the initial 24 h, and is highly influenced by the quantitative extension of calcium hydroxide-induced alkalinity and Ca^2+^ release [27,31]. In fact, the superior precipitation properties could be accredited to the HCSCs that produce a higher amount of calcium hydroxide during the initial hydration reaction or have a supplemental calcium hydroxide added to the composition as seen in TF and TFHF. The dissolved calcium hydroxide creates a favorable environment for the nucleation of calcium phosphate, which gradually fills the empty spaces and results in a substantial reduction in overall porosity [25]. Therefore, considering the fact that less porous materials may present more available atoms to interact with the incident X-rays [32], the significantly increased long-term radiopacity, exhibited by TFHF, TF, and BR, may be directly linked to a more efficient precipitation process and thus a more densely packed material matrix with lower degradation and solubility rates [25,33]. 

The present study suggests that all the tested HCSCs have a time-dependent tendency to increase the radiopacity. Even though there are still no clear data on how susceptible human eyes are to detecting these radiopacity changes clinically, the filling material may theoretically appear more homogeneous and uniform on the follow-up radiographs. Nevertheless, it must be highlighted that radiopacity changes are strongly related to the environmental conditions that may affect many important characteristics of HCSCs, including the previously mentioned setting process and surface structure [34,35]. The current guidelines indicate that a widely applied immersion of HCSCs into the storage solution is not the best model to stimulate clinical conditions, as HCSCs are sensitive to moisture and may exhibit a high solubility before the final setting [31]. For this reason, the gelatinized HBSS solution was used in the present study, aiming to imitate an in vivo environment and avoid material washout. However, no evidence-based information has been published to date regarding the amount of moisture that HCSCs may receive from the pulp or surrounding periodontal tissues at early and later post-operative stages. Moreover, the HBSS storage medium is not capable of fully reflecting the complexity of the physiological fluids and the individual effects on their components. It impedes the precise reproducibility of clinical situations by simplified in vitro models, and thus the results of the present study should be evaluated with the interpretation that HCSCs have the potential to increase their radiopacity with time under favorable environmental conditions. 

The available scientific data confirm that clinical radiopacity may also interfere with various factors, including exposure parameters, overlapping bone and dental tissues, X-ray imaging systems and techniques, source-to-object distance, angulation, etc. [36]. On account of these observations, radiopacity values obtained in the present study should be considered as indicative estimates only, which enable the comparative analysis of commonly used HCSCs and thus lead to the conclusion of AHPB being the most radiopaque tested material. It can be noticed that clinicians usually prefer the use of highly radiopaque endodontic materials in order to obtain a more favorable post-operative radiological view. However, the excessive radiopacity may compromise the diagnostic accuracy of radiographs or CBCT due to the presence of beam-hardening artefacts, which occur as a result of X-ray penetration through the highly dense area [37]. On the other hand, less radiopaque HCSCs, e.g., BD, may provide a false-negative interpretation of tightly packed fillings and even be considered as absent in some areas. Therefore, it would be highly valuable to determine and confirm the optimal radiopacity level for all endodontic filling materials. However, there is no such provision to date, and thus, clinicians have to make their own decisions about what filling material to use in daily clinical practice.

## 5. Conclusions

The radiographic analysis revealed that all the tested HCSCs exceeded the ISO-specified limit of 3 mm Al, with AHPB being the most radiopaque material and BD the least. Significant radiopacity changes were observed only for BR, TFHF, and TF after 28 days in storage media, whereas none of the tested materials demonstrated considerable variances between 30 min and 24 h radiopacity values. However, all the tested materials had a time-dependent tendency to increase the radiopacity, along with precipitate formation on the surface, leading to considerations that HCSCs have a potential to increase their radiopacity with time under favorable environmental conditions due to particle rearrangement and deposition of hydration products.

## Figures and Tables

**Table 1:** Composition and preparation information of tested HCSCs.

Material	Composition Declared by Manufacturers	Preparation
AH Plus Bioceramic Sealer (AHPB)	Tricalcium silicate, lithium carbonate, zirconium oxide, dimethyl sulfoxide, thickening agents	Paste ready to use
Bio-C Sealer (BIOC)	Tricalcium silicate, dicalcium silicate, tricalcium aluminate, calcium oxide, zirconium oxide, silicon oxide, iron oxide, polyethylene glycol	Paste ready to use
Biodentine (BD)	Powder: tricalcium silicate, dicalcium silicate, calcium carbonate, calcium oxide, iron oxide, zirconium oxide Liquid: water, calcium chloride, polycarboxylate	1 capsule of BD to 5 drops of liquid mixed for 30 s in the amalgamator
BioRoot RCS (BR)	Powder: tricalcium silicate, zirconium oxide, povidone Liquid: water, calcium chloride, polycarboxylate	1 spoon of powder to 5 drops of liquid mixed for 60 s until a smooth paste
Grey-MTAFlow (GMF)	Powder: tricalcium silicate, dicalcium silicate, calcium sulfate, silica, bismuth oxide Liquid: water, water-soluble silicone-based gel	1 big-end plus 1 small-end spoon of powder (0.19 g) to 3 drops of liquid mixed until a thin consistency
White-MTAFlow (WMF)	Powder: tricalcium silicate, dicalcium silicate, calcium sulfate, tantalum oxide Liquid: water, water-soluble silicone-based gel	1 big-end plus 1 small-end spoon of powder (0.19 g) to 3 drops of liquid mixed until a thin consistency
TotalFill BC Sealer (TF)	Tricalcium silicate, dicalcium silicate, calcium phosphate monobasic, zirconium oxide, tantalum oxide, calcium hydroxide, filler and thickening agents	Paste ready to use
TotalFill BC Sealer HiFlow (TFHF)	Tricalcium silicate, dicalcium silicate, calcium hydroxide, zirconium oxide, filler and thickening agents	Paste ready to use

**Table 2:** Mean values and standard deviations of radiopacity at different time moments.

Material	Radiopacity (mm Al)			
	30 min	24 h	28 days	Total Increase
AHPB	10.82 ± 0.69	11.07 ± 0.94	11.26 ± 0.65	0.44
BIOC	8.15 ± 0.44	8.17 ± 0.41	8.85 ± 0.44	0.70
BD	3.34 ± 0.43	3.35 ± 0.40	3.75 ± 0.36	0.41
BR	7.19 ± 0.32 ^A^	7.47 ± 0.35 ^B^	8.08 ± 0.40 ^A,B^	0.89
GMF	6.27 ± 0.41	6.62 ± 0.37	6.98 ± 0.32	0.71
WMF	5.76 ± 0.20	5.80 ± 0.48	6.20 ± 0.46	0.44
TF	8.56 ± 0.47 ^C^	9.03 ± 0.25	9.66 ± 0.73 ^C^	1.10
TFHF	8.81 ± 0.30 ^D^	9.19 ± 0.31 ^E^	9.79 ± 0.43 ^D,E^	0.98

The same superscript letter in the line indicates statistically significant differences between radiopacity values (*p* < 0.05).

## Data Availability

Data are contained within the article.
